# An Adaptive Weight Method for Image Retrieval Based Multi-Feature Fusion

**DOI:** 10.3390/e20080577

**Published:** 2018-08-06

**Authors:** Xiaojun Lu, Jiaojuan Wang, Xiang Li, Mei Yang, Xiangde Zhang

**Affiliations:** College of Sciences, Northeastern University, Shenyang 110819, China

**Keywords:** image retrieval, multi-feature fusion, entropy, relevance feedback

## Abstract

With the rapid development of information storage technology and the spread of the Internet, large capacity image databases that contain different contents in the images are generated. It becomes imperative to establish an automatic and efficient image retrieval system. This paper proposes a novel adaptive weighting method based on entropy theory and relevance feedback. Firstly, we obtain single feature trust by relevance feedback (supervised) or entropy (unsupervised). Then, we construct a transfer matrix based on trust. Finally, based on the transfer matrix, we get the weight of single feature through several iterations. It has three outstanding advantages: (1) The retrieval system combines the performance of multiple features and has better retrieval accuracy and generalization ability than single feature retrieval system; (2) In each query, the weight of a single feature is updated dynamically with the query image, which makes the retrieval system make full use of the performance of several single features; (3) The method can be applied in two cases: supervised and unsupervised. The experimental results show that our method significantly outperforms the previous approaches. The top 20 retrieval accuracy is 97.09%, 92.85%, and 94.42% on the dataset of Wang, UC Merced Land Use, and RSSCN7, respectively. The Mean Average Precision is 88.45% on the dataset of Holidays.

## 1. Introduction

As an important carrier of information, it is significant to do efficient research with images [1,2,3,4,5,6]. Large-scale image retrieval has vast applications in many domains such as image analysis, search of image over internet, medical image retrieval, remote sensing, and video surveillance [7,8,9,10,11,12,13,14,15,16,17,18,19,20,21,22,23,24]. There are two common image retrieval systems: text-based image retrieval system and content-based image retrieval system. Text-based image retrieval system requires experienced experts to mark images, which is very expensive and time-consuming [7]. Content-based retrieval systems can be divided into two categories [8]. One is based on global features indexed with hashing strategies; another is local scale invariant features indexed by a vocabulary tree or a k-d tree. The two characteristics have pros and cons, and their performance complements each other [6,8]. In recent years, many excellent works focused on improving the accuracy and efficiency have been done [6]. A dynamically updating Adaptive Weights Allocation Algorithm (AWAA) which rationally allocates fusion weights proportional to their contributions to matching is proposed previously [7], which helps ours gain more complementary and helpful image information during feature fusion. In a previous paper [8], the authors improve reciprocal neighbor based graph fusion approach for feature fusion by the SVM prediction strategy, which increases the robustness of original graph fusion approach. In another past paper [9], the authors propose a graph-based query specific fusion approach where multiple retrieval sets are merged and are reranked by conducting a link analysis on a fused graph, which is capable of adaptively integrating the strengths of the retrieval methods using local or holistic features for different queries without any supervision. In a previous paper [10], the authors propose a simple yet effective late fusion method at score level by score curve and weighting different features in a query-adaptive manner. In another previous paper [11], the authors present a novel framework for color image retrieval through combining the ranking results of the different descriptors through various post-classification methods. In a past work [12], the authors propose robust discriminative extreme learning machine (RDELM), which enhances the discrimination capacity of ELM for RF. In a previous paper [13], the authors present a novel visual word integration of Scale Invariant Feature Transform (SIFT) and Speeded-Up Robust Features (SURF). The visual words integration of SIFT and SURF adds the robustness of both features to image retrieval. In another past work [14], an improved algorithm for center adjustment of RBFNNs and a novel algorithm for width determination have been proposed to optimize the efficiency of the Optimum Steepest Decent (OSD) algorithm, which achieves fast convergence speed, better and same network response in fewer train data. In a previous paper [15], an edge orientation difference histogram (EODH) descriptor and image retrieval system based on EODH and Color-SIFT was shown. In a previous paper [16], the authors investigate the late fusion of FREAK and SIFT to enhance the performance of image retrieval. In a previous paper [17], the authors propose to compress the CNN features using PCA and obtain a good performance. In a previous paper [18], the authors improve recent methods for large scale image search, which includes introducing a graph-structured quantizer and using binary. 

Although the above methods have achieved good results, the performance of the retrieval system still has much room for improvement. In order to improve the performance of the retrieval system, it is an effective strategy to integrate multiple features for image retrieval [19,20,21,22,23,24,25,26,27]. Measurement level fusion is widely used, but how to determine the weight of each feature to improve the retrieval performance is still a very important problem [10,20,28]. In a previous paper [20], the author uses average global weight to fuse Color and Texture features for image retrieval. In a previous paper [9], the authors propose a graph-based query specific fusion approach without any supervision. In a previous paper [10], the author uses the area under the score curve of retrieval based on a single feature as the weight of the feature. The performances of different weight determination methods are different. The adaptive weights can achieve better retrieval performance than the global weights. In order to further improve the performance of the retrieval system, unlike previous weight determination methods, this paper proposes a new adaptive weight determination method based on relevance feedback and entropy theory to fuse multiple features. Our method has three outstanding advantages. (1) The retrieval system combines the performance of multiple features and has better retrieval accuracy and generalization ability than single feature retrieval system; (2) In each query, the weight of a single feature is updated dynamically with the query image, which makes the retrieval system make full use of the performance of several single features; (3) Unsupervised image retrieval means that there is no manual participation in the retrieval process. In an image search, no supervision is more popular than supervision. If we pursue higher retrieval accuracy, supervision is necessary. But from the perspective of user experience, unsupervised is better. It is worth mentioning that the method can be applied in two cases: supervised and unsupervised. Getting our method, firstly, we obtain single feature trust based on relevance feedback (supervised) or entropy (unsupervised); next, we construct a transfer matrix based on trust; finally, based on the transfer matrix, we get the weight of single feature through several iterations, which makes full use of single feature information of image and can achieve higher retrieval accuracy.

## 2. Related Work

For the image retrieval system integrating multi-features at measurement level, this paper mainly focus on how to determine the weight of each feature to improve the retrieval accuracy. In this section, we mainly introduce some work related to our method.

### 2.1. Framework

The main process of common system framework for image retrieval based on fusion of multiple features at the metric level is as follows [28,29,30,31,32]. Firstly, we extract several features of image and build benchmark image database. Then, when users enter images, we calculate the similarity between the query image and images of the database based on several features, separately. Finally, we get the comprehensive similarity measure by weighting several similarities and output retrieval results based on it. 

### 2.2. The Ways to Determine Weight

A lot of work has been done to improve the performance of the retrieval system with multiple features [33,34]. At present, feature fusion is mainly carried out on three levels [8]: feature level, index level, and sorting level. The method proposed in this paper is applicable to the fusion of measurement level. Traditionally, there are two ways to determine the weight of feature, the global weight [11,20,32], and the adaptive weight [10,35], the pros/cons of each are listed in Table 1. The former is reciprocal of the number of features or decided by experienced experts, which leads the retrieval system to have poor generalization performance and low retrieval performance for different retrieval images. The latter is derived from retrieval feedback based on this feature, which is better than the global weight. However, in the sum or product fusion, the distinction between good features and bad features, is not obvious. If the weights of the bad features in the retrieval work are large, it will also reduce the retrieval performance to a certain extent. In order to clearly distinguish good features and bad features and the retrieval system can make full use of their performance to achieve better retrieval accuracy, a new adaptive weight retrieval system is proposed. Firstly, we obtain single feature trust based on relevance feedback (supervised) or entropy (unsupervised). Next, we construct a transfer matrix based on trust. Finally, based on the transfer matrix, we get the weight of single feature through several iterations, which makes full use of single feature information of image, and can achieve higher retrieval accuracy. 

The common weighted fusion methods of measurement level are maximum fusion, multiplication fusion [10], and sum fusion [11,32]. The comprehensive metric obtained by maximum fusion is obtained from the feature with the maximum weight. The comprehensive metric obtained by multiplication fusion is the product of different weighted similarity measures. The comprehensive metric obtained by sum fusion is the adding of different weighted similarity measures. Specifically, K features labeled as are fused, *q* is a query image, $p_{k}\in \left\{p_{1},p_{2},\ldots ,p_{n}\right\}$ is a target image of database $\Omega =\left\{p_{1},p_{2},\ldots ,p_{n}\right\}$. Each method of fusion is shown as follows:

The maximum fusion:

(1) $$sim(q)=\underset{D_{i}(q)}{\arg}\;\max\{w_{q}{}^{\left(i\right)}|i=1,2,\ldots \ldots ,K\}\;$$

The multiplication fusion:

(2) $$sim(q)=\prod_{i=1}^{K}w_{q}{}^{\left(i\right)}D_{i}(q),\;\{i=1,2,\ldots \ldots ,K\}\;$$

The multiplication fusion:

(3) $$sim(q)=\sum_{i=1}^{K}w_{q}{}^{\left(i\right)}D_{i}(q),\;\{i=1,2,\ldots \ldots ,K\}\;$$

Here, q is a query image. K is the number of feature. $w_{q}{}^{i}$ is weight of $F_{i}\in \left\{F_{1},F_{2},\ldots ,F_{K}\right\}$. $D_{i}(q)\in \left\{D_{1}(q),D_{2}(q),\ldots ,D_{K}(q)\right\}$ is the similarity vector between the query image *q* and images of database $\Omega =\left\{p_{1},p_{2},\ldots ,p_{n}\right\}$, which is calculated based on feature $F_{i}\in \left\{F_{1},F_{2},\ldots ,F_{K}\right\}$. sim(q) is Comprehensive similarity measure. 

### 2.3. Relevance Feedback

The relevance feedback algorithm [34] is used to solve the semantic gap problem in content-based image retrieval, and the results obtained by relevance feedback are very similar to those of human [36,37]. The main steps of relevance feedback are as follows: first, the retrieval system provides primary retrieval results according to the retrieval keys provided by the user; then, the user determines which retrieval results are pleasant; finally, the system then provides new retrieval results according to the user’s feedback. In this paper, we get the trust of single feature under the supervised condition through relevance feedback. Under the condition of supervision, this paper obtains the trust of single feature through relevance feedback. 

## 3. Proposed Method

In this section, we will introduce our framework and adaptive weight strategy. 

### 3.1. Our Framework

**Figure 1:**
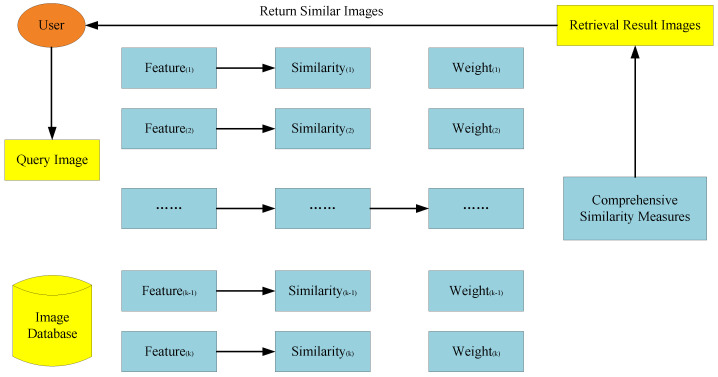
The proposed retrieval system framework.

For a specific retrieval system, the weight of each feature is static in different queries. It causes low retrieval performance. In order to overcome the shortcoming, a new image retrieval system based on multi-feature is proposed. The basic framework of the retrieval system is shown in Figure 1. 

In the database creation phase, firstly, we extract features separately; then, we calculate the entropy of different feature dimensions based on each feature; finally, we save features and entropies to get the image feature database. The original image database is a collection of large numbers of images. The established image feature database and the original image database are in a one-to-one correspondence, for example, the image 1.jpg is stored in the image database. The storage form of the image feature database is 1.jpg (image name), feature, and entropy. In this paper, what we call an image database is actually an image feature database. 

In the image search phase, when users enter images, firstly, we calculate the similarity between the query image and the images of database based on each feature separately; then, we get the trust of a single feature; finally, we get the comprehensive similarity measure by weighting several measures and output retrieval results based on it. 

Specifically, K features labeled as are fused, q is a query image, $p_{k}\in \left\{p_{1},p_{2},\ldots ,p_{n}\right\}$ is a target image of database $\Omega =\left\{p_{1},p_{2},\ldots ,p_{n}\right\}$. The proposed fusion method is as follows. 

Firstly, considering that it will take a long time to calculate similarity measures using several features, we get binary feature as follows:

For each bit of feature $F_{i}\in \left\{F_{1},F_{2},\ldots ,F_{K}\right\}$, we output binary codes $F_{i}\in \left\{F_{1},F_{2},\ldots ,F_{K}\right\}$ by:

(4) $$ave(F_{i})=\frac{\sum_{j=1}^{m}F_{i}(c_{j})}{m}\;$$

(5) $$F_{i}(c_{j})=\left\{ \begin{array}{l} 1\;F_{i}(c_{j})\geq ave(F_{i})\\ 0\;F_{i}(c_{j})<ave(F_{i}) \end{array}\right.\;i\in \left\{1,2,\ldots ,K\right\}\;$$

Here, $ave(F_{i})$ is the mean of feature $F_{i}\in \left\{F_{1},F_{2},\ldots ,F_{K}\right\}$, m is the dimension of feature $F_{i}\in \left\{F_{1},F_{2},\ldots ,F_{K}\right\}$, $F_{i}(c_{j})$ is the *j*-th component of feature $F_{i}\in \left\{F_{1},F_{2},\ldots ,F_{K}\right\}$. 

Then, we calculate the distance between *q* and *p*, then normalize it:

(6) $$d^{i}(k)=d^{i}(q,p_{k})=\sum_{j=1}^{m}w_{j}\left|Fq^{i}(j)-Fp_{k}{}^{i}(j)\right|\;k\in \left\{1,2,\ldots ,n\right\},i\in \{1,2,\ldots ,K\}\;$$

(7) $$D_{i}(q)=1-\frac{1}{\sum_{k=1}^{n}d^{i}(k)}(d^{i}(1),d^{i}(2),\ldots ,d^{i}(n))\;(i\in \left\{1,2,\ldots ,K\right\})\;$$

Here, $D_{i}(q)\in \left\{D_{1}(q),D_{2}(q),\ldots ,D_{K}(q)\right\}$ is the similarity vector between the query image *q* and images of database $\Omega =\left\{p_{1},p_{2},\ldots ,p_{n}\right\}$, which is calculated based on feature $F_{i}\in \left\{F_{1},F_{2},\ldots ,F_{K}\right\}$. n is the total number of images. $Fq^{i}$, $Fp_{k}{}^{i}$ respectively represent the feature $F_{i}\in \left\{F_{1},F_{2},\ldots ,F_{K}\right\}$ of *q* and of $p_{k}\in \left\{p_{1},p_{2},\ldots ,p_{n}\right\}$. 

We calculate the comprehensive measure sim(q) by fusing multiple features:

(8) $$sim(q)=\sum_{i=1}^{K_{1}}{\tilde{w}}_{q}^{\left(i\right)}D_{i}(q)+\sum_{i=K_{1}+1}^{K}w_{q}{}^{\left(i\right)}D_{i}(q)\;$$

Here $\tilde{w_{q}{}^{\left(i\right)}}\in \left\{\tilde{w_{q}{}^{\left(1\right)}},\tilde{w_{q}{}^{\left(2\right)}},\ldots ,\tilde{w_{q}{}^{\left(K_{1}\right)}}\right\}$, $w_{q}{}^{\left(i\right)}\in \left\{w_{q}{}^{\left(K_{1}+1\right)},w_{q}{}^{\left(K_{1}+2\right)},\ldots ,w_{q}{}^{\left(K\right)}\right\}$, $K_{1}$ are the weight of a good feature, the weight of a bad feature, and the number of good features, respectively.

Finally, we sort the similarity sim(q) and get the final search results. 

### 3.2. Entropy of Feature

Information entropy is the expected value of the information contained in each message [38], represented as (n is the number of messages):

(9) $$H_{\left(x\right)}=E(I_{\left(x\right)})=\sum_{j=1}^{N}p(x)\log_{2}p{\left(x\right)}^{-1}\;$$

Here, *X* is a random phenomenon. *X* contains *N* possibility. p(x) is the probability of *x*. H(X) is the nondeterminacy of the occurrence of *X*.

In our work, the entropy of *j*-th dimension feature is calculated as follows:

(10) $$H_{j}=-\frac{1}{\log_{2}n}\sum_{i=1}^{n}\frac{f_{ij}}{\sum_{i=1}^{n}f_{ij}}\;\log_{{}_{2}}\frac{\sum_{i=1}^{n}f_{ij}}{f_{ij}}\;,\;j\in \left\{1,2,\ldots ,m\right\}\;$$

Here, *N* is the number of images in the database. *M* is the feature dimension. $f_{ij},i\in \left\{1,2,\ldots ,n\right\}$, j∈{1,2,…,m} is the *j*-th dimension feature of *i*-th image. 

The weights of *j*-th dimension is calculated as follows:

(11) $$w_{j}=\frac{e^{\left(1-H_{j}\right)}}{\sum_{j=1}^{m}e^{\left(1-H_{j}\right)}}\;,\;j\in \left\{1,2,\ldots ,m\right\}\;$$

Here, $H_{j}$ is the entropy of *j*-th dimension feature. $w_{j}$ is the weight of *j*-th dimension.

When all the values of feature are equal, the entropy $H_{j}$ is 1. The weight of each feature component is equal to $\frac{1}{m}\;$.

### 3.3. Adaptive Weight Strategy

To overcome the problem of low retrieval performance caused by the weight determination method used with multiple feature fusion, this paper proposes a new method to obtain single feature weight. Our method can be applied to supervised learning and unsupervised learning. The specific methods are as follows:

Under the circumstances of supervision, the weight of a single feature is obtained based Relevance Feedback. $D_{i}(q)\in \left\{D_{1}(q),D_{2}(q),\ldots ,D_{K}(q)\right\}$ is the similarity vector between the query image *q* and images of database, which is calculated based on feature $F_{i}\in \left\{F_{1},F_{2},\ldots ,F_{K}\right\}$. We sort $D_{i}(q)\in \left\{D_{1}(q),D_{2}(q),\ldots ,D_{K}(q)\right\}$ and return search results by it. The results are labeled as $a^{i}=\{a_{1}{}^{i},a_{2}{}^{i},\ldots \ldots ,a_{t}{}^{i}\}$. Here, t represents the predefined number of returned images. The retrieved results are evaluated according to relevant feedback. The $pre_{x},pre_{y}\in \left\{pre_{1},pre_{2},\ldots ,pre_{K}\right\}$ as trust of single feature retrieval is calculated. That is to say, we rely on the feedback to evaluate the retrieval results, and then use the evaluation index on the dataset to calculate the retrieval performance that is the trust of the feature. For example, on the Wang dataset with the precision as the evaluation index, we search images based on $F_{i}\in \left\{F_{1},F_{2},\ldots ,F_{K}\right\}$. If we find have h1 similar images in the h retrieval results by relevant feedback, we believe the trust of $F_{i}\in \left\{F_{1},F_{2},\ldots ,F_{K}\right\}$ is h1/h. By several iterations, the weight of single feature is as follows: firstly, we structure the transfer matrix $H_{kk}=\{H(x,y)\}$, representing the performance preference among each feature. Note that the feature $F_{x}\in \left\{F_{1},F_{2},\ldots ,F_{K}\right\}$ goes to feature $F_{y}\in \left\{F_{1},F_{2},\ldots ,F_{K}\right\}$ with a bias of H(x,y), the detailed construction process of $H_{KK}=\{H(x,y)\}$ is as follows:

$$\begin{array}{l} if\;pre_{y}>=pre_{x}\\ \;H(x,y)=e^{\alpha (pre_{y}-pre_{x})}\text{​ }(\alpha \geq 1)\\ else\;\\ \;H(x,y)=\left|pre_{y}-pre_{x}\right| \end{array}\;$$

When the trust of $F_{y}\in \left\{F_{1},F_{2},\ldots ,F_{K}\right\}$ is greater than $F_{x}\in \left\{F_{1},F_{2},\ldots ,F_{K}\right\}$, in order to obtain better retrieval result, we believe that $F_{x}\in \left\{F_{1},F_{2},\ldots ,F_{K}\right\}$ can be replaced by $F_{y}\in \left\{F_{1},F_{2},\ldots ,F_{K}\right\}$. The replacement depends on the parameter α. The larger α is, the more the retrieval system depends on $F_{y}\in \left\{F_{1},F_{2},\ldots ,F_{K}\right\}$. The α≥1 is because $F_{y}\in \left\{F_{1},F_{2},\ldots ,F_{K}\right\}$ is better than $F_{x}\in \left\{F_{1},F_{2},\ldots ,F_{K}\right\}$, we need to get $e^{\alpha (pre_{y}-pre_{x})}\text{​}>\left|pre_{y}-pre_{x}\right|$, so that the weight of $F_{y}\in \left\{F_{1},F_{2},\ldots ,F_{K}\right\}$ is larger and retrieval system relies more on $F_{y}\in \left\{F_{1},F_{2},\ldots ,F_{K}\right\}$. When the trust of $F_{y}\in \left\{F_{1},F_{2},\ldots ,F_{K}\right\}$ is equal to $F_{x}\in \left\{F_{1},F_{2},\ldots ,F_{K}\right\}$, we believe that the $F_{x}\in \left\{F_{1},F_{2},\ldots ,F_{K}\right\}$ can be replaced by $F_{y}\in \left\{F_{1},F_{2},\ldots ,F_{K}\right\}$ the replacement bias H(x,y) is 1. When the trust of $F_{y}\in \left\{F_{1},F_{2},\ldots ,F_{K}\right\}$ is less than $F_{x}\in \left\{F_{1},F_{2},\ldots ,F_{K}\right\}$, we think that $F_{x}\in \left\{F_{1},F_{2},\ldots ,F_{K}\right\}$ can still be replaced by $F_{y}\in \left\{F_{1},F_{2},\ldots ,F_{K}\right\}$, but the replacement bias H(x,y) is relatively small. One benefit is that although retrieval performance based on some of the features of image retrieval is poor, we still believe that it is helpful for the retrieval task. 

Then, the weight of a single feature is obtained by using the preference matrix. We initialize the weight $w_{1}$ to $w_{1}=\left\{\frac{1}{K},\frac{1}{K},\ldots ,\frac{1}{K}\right\}$. $w=\{w_{F_{1}},w_{F_{2}},\ldots ,w_{F_{K}}\}$ is the weight of a single feature. The $w_{d}$ is the newly acquired weights through iterations. The $w_{d-1}$ is the weight of the previous iteration. We use the transfer matrix $H_{KK}=\{H(x,y)\}$ to iterate the weights based on formula 12.

(12) $${\left[\begin{array}{l} w^{\prime }{}_{F_{1}}\\ w^{\prime }{}_{F_{2}}\\ \;\vdots \\ w^{\prime }{}_{F_{K}} \end{array}\right]}_{\left(d\right)}=\gamma w^{\prime }{}_{d-1}{\left[\begin{array}{l} w^{\prime }{}_{F_{1}}\\ w^{\prime }{}_{F_{2}}\\ \;\vdots \\ w^{\prime }{}_{F_{K}} \end{array}\right]}_{\left(d-1\right)}+(1-r)[\begin{array}{l} H(F_{1},F_{2})\;\cdots \\ H(F_{2},F_{1})\;\cdots \\ \;\vdots \\ H(F_{K},F_{1})\;\cdots \end{array}\;\begin{array}{l} H(F_{1},F_{K})\\ H(F_{2},F_{K})\\ \;\vdots \\ H(F_{K},F_{K}) \end{array}]{\left[\begin{array}{l} w^{\prime }{}_{F_{1}}\\ w^{\prime }{}_{F_{2}}\\ \;\vdots \\ w^{\prime }{}_{F_{K}} \end{array}\right]}_{\left(d-1\right)},(\gamma \in [0,1])\;$$

The $w_{d}$ depends not only on the choice of features depending on the transfer matrix, but also on the $w_{d-1}$ obtained from the previous calculation. The degree of dependence on the above two depends on the parameter γ. An obvious advantage of this voting mechanism is that it will not affect the final result because of a relatively poor decision. The process is as follows:

$$\begin{array}{l} w_{d}=\left\{\frac{1}{K},\frac{1}{K},\ldots ,\frac{1}{K}\right\},\;d=1\\ repeat\\ \;w^{\prime }{}_{d}=\gamma w^{\prime }{}_{d-1}+(1-r)HHw^{\prime }{}_{d-1}\;(\gamma \in [0,1])\\ \;w_{d}=w_{d}/sum(w_{d})\\ \;d\leftarrow d+1\\ Until\;\left\|w_{d}-w_{d-1}\right\|<\varepsilon \;(\varepsilon \geq 0)\\ return\;w_{d} \end{array}\;$$

● Good features and bad features

In our method, the weight of a single feature is different for different queries. In order to improve the retrieval accuracy, we hope that the features with better retrieval performance can have larger weight than those with poor retrieval performance. For this reason, we divide features into good features and bad features according to retrieval performance. We search image based on $F_{y}\in \left\{F_{1},F_{2},\ldots ,F_{K}\right\}$ and $F_{x}\in \left\{F_{1},F_{2},\ldots ,F_{K}\right\}$, respectively. If the retrieval performance of $F_{y}\in \left\{F_{1},F_{2},\ldots ,F_{K}\right\}$ is better than $F_{x}\in \left\{F_{1},F_{2},\ldots ,F_{K}\right\}$, we think that $F_{y}\in \left\{F_{1},F_{2},\ldots ,F_{K}\right\}$ is a good feature and $F_{x}\in \left\{F_{1},F_{2},\ldots ,F_{K}\right\}$ is a bad feature. Good features and bad features are specifically defined as follows:

(13) $$\begin{array}{l} \;if\;pre_{y}>=pre_{x}\\ \;pre_{y}\in \{good\_feature\}\\ else\\ \;pre_{x}\in \{bad\_feature\} \end{array}\;$$

Here, $pre_{y}\in \left\{pre_{1},pre_{2},\ldots ,pre_{K}\right\}$ is the retrieval performance of $F_{y}\in \left\{F_{1},F_{2},\ldots ,F_{K}\right\}$, $pre_{x}\in \left\{pre_{1},pre_{2},\ldots ,pre_{K}\right\}$ is the retrieval performance of $F_{x}\in \left\{F_{1},F_{2},\ldots ,F_{K}\right\}$. 

● Our method for unsupervised

Image retrieval based on the above adaptive weight strategy is a supervised retrieval process and users need to participate in the feedback of single feature trust. In the actual application process, users may prefer the automatic retrieval system. That is to say, unsupervised retrieval system without manual participation is more popular. Therefore, considering the advantages of unsupervised image retrieval, we further study this method and propose an adaptive weight method under unsupervised conditions. The unsupervised method is basically the same as the supervised method. The only difference is, in contrast to the supervised process, the weight of a single feature is obtained based entropy rather than relevant feedback. 

First, the entropy of $D_{i}(q)=(d^{*i}(1),d^{*i}(2),\ldots \ldots ,d^{*i}(n))$ is:

(14) $$H_{i}=-\frac{1}{\log_{2}{}^{n}}\sum_{j=1}^{n}\frac{d^{*i}(j)}{\sum_{j=1}^{n}d^{*i}(j)}\;\log_{2}\left(\frac{\sum_{j=1}^{n}d^{*i}(j)}{d^{*i}(j)}\right)\;,\;i\in \left\{1,2,\ldots ,k\right\}\;$$

Here, $D_{i}(q)\in \left\{D_{1}(q),D_{2}(q),\ldots ,D_{K}(q)\right\}$ is the similarity vector between the query image *q* and images of database, which is calculated based on feature $F_{i}\in \left\{F_{1},F_{2},\ldots ,F_{K}\right\}$. n is the total number of images. $d^{*i}(j)$ is the similarity between the query image *q* and *j*-th image of database.

Then, the trust of $D_{i}(q)\in \left\{D_{1}(q),D_{2}(q),\ldots ,D_{K}(q)\right\}$ is:

(15) $$pre_{i}=H_{i}\;$$

Here, $D_{i}(q)\in \left\{D_{1}(q),D_{2}(q),\ldots ,D_{K}(q)\right\}$ is the similarity vector between the query image *q* and images of database, which is calculated based on feature $F_{i}\in \left\{F_{1},F_{2},\ldots ,F_{K}\right\}$. $pre_{i}\in \left\{pre_{1},pre_{2},\ldots ,pre_{K}\right\}$ is the retrieval performance of $F_{i}\in \left\{F_{1},F_{2},\ldots ,F_{K}\right\}$. 

After gaining trust, the weight seeking process is the same as the supervised state.

## 4. Performance Evaluation

### 4.1. Features

The features we choose in this article are as follows:

- Color features. For each image, we compute 2000-dim HSV histogram (H, S, and V are 20, 10, and 10).
- CNN-feature1. The model we used to get CNN feature is VGG-16 [39]. We directly use pre-trained models to extract features from the fc7 layer as CNN features.
- CNN-feature2. The model we used to get CNN feature is AlexNet which is pre-trained by Simon, M., Rodner, E., Denzler, J., in their previous work [40]. We directly use the model to extract features from the fc7 layer as CNN features. The dimension of the feature is 4096.

The extraction methods of color feature, cnn-feature1, and cnn-feature2 belong to the results of the original papers and are well-known. So we did not retell it. However, the feature extraction code we adopted has been shared to the website at https://github.com/wangjiaojuan/An-adaptive-weight-method-for-image-retrieval-based-multi-feature-fusion.

### 4.2. Database and Evaluation Standard

- Wang (Corel 1K) [41]. That contains 1000 images that are divided into 10 categories. The precision of Top-r images is used as the evaluation standard of the retrieval system.
- Holidays [42]. That includes 1491 personal holiday pictures and is composed of 500 categories. mAp is used to evaluate the retrieval performance.
- UC Merced Land Use [43]. That contains 21 categories. Each category has 100 remote sensing images. Each image is taken as query in turn. The precision of Top-r images is used as the evaluation standard of the retrieval system.
- RSSCN7 [44]. That contains 2800 images which are divided into 7 categories. Each category has 400 images. Each image is taken as query in turn. The precision of Top-r images is used as the evaluation standard of the retrieval system.

The precision of Top-r images is calculated as follows:

(16) $$precision=\frac{N_{r}}{r}\;$$

Here, $N_{r}$ is the number of relevant images matching to the query image, r is the total number of results returned by the retrieval system. 

The mAp is calculated as follows:

(17) $$mAp=\frac{1}{\left|Q\right|}\sum_{i=1}^{\left|Q\right|}\frac{1}{RN_{i}}\sum_{j=1}^{RN_{i}}P(RS^{j}{}_{i})\;$$

Here, |Q| is the number of query images, suppose $q_{i}\in Q$ is a retrieval image, $RN_{i}$ is the total number of relevant images matching to $q_{i}$, $RS^{j}{}_{i}$ is $RS^{j}{}_{i}\_th$ similar image of query result and $NR^{j}{}_{i}$ is location information, $P(RS^{j}{}_{i})$ is the evaluation of retrieval results of $q_{i}$ and is calculated as follows:

(18) $$P(RS^{j}{}_{i})=\frac{RS^{j}{}_{i}}{NR^{j}{}_{i}}\;$$

### 4.3. Evaluation of the Effectiveness of Our Method

The main innovations of our method are as follows. (1) Based on entropy, we weigh features to improve the accuracy of similarity measurement; (2) Under the supervised condition, we obtain the single feature weight based on related feedback and fuse multi-feature at the measurement level to improve the retrieval precision; (3) Under the unsupervised condition, we obtain the single feature weight based on entropy and fuse multiple features at the measurement level to improve the retrieval precision. To verify the effectiveness of the method, we carried out experiments on Holidays, Wang, UC Merced Land Use, and RSSCN7.

We have done the following experiments. (1) Retrieve image based on CNN1-feature, Color feature, and CNN2-feature, respectively. At the same time, experiments are carried out under two conditions: entropy and no entropy; (2) under the state of supervision, retrieve image by fusing three different features which respectively uses relevance feedback and our method; (3) under the state of unsupervision, retrieve image by fusing three different features which respectively uses average global weights and our method. An implementation of the code is available at https://github.com/wangjiaojuan/An-adaptive-weight-method-for-image-retrieval-based-multi-feature-fusion.

#### 4.3.1. Unsupervised

Under the unsupervised condition, in order to verify the effectiveness of the adaptive weight method proposed in this paper, we carried out experiments on Holidays, Wang, UC Merced Land Use, and RSSCN7 datasets. Table 2 shows a comparison of retrieval results based on AVGand OURS. On the Holidays dataset, our method is better than RF, and improves the retrieval precision by 5.12%. On the Wang dataset, our method improves the retrieval accuracy by 0.35% (Top 20), 0.47% (Top 30), and 0.58% (Top 50) compared with AVG. On the UC Merced Land Use dataset, our method improves the retrieval accuracy by 6.61% (Top 20), 9.33% (Top 30), and 12.59% (Top 50) compared with AVG. On the RSSCN7 dataset, our method improves the retrieval accuracy by 2.61% (Top 20), 3.14% (Top 30), and 3.84% (Top 50) compared with AVG.

**Figure 2:**
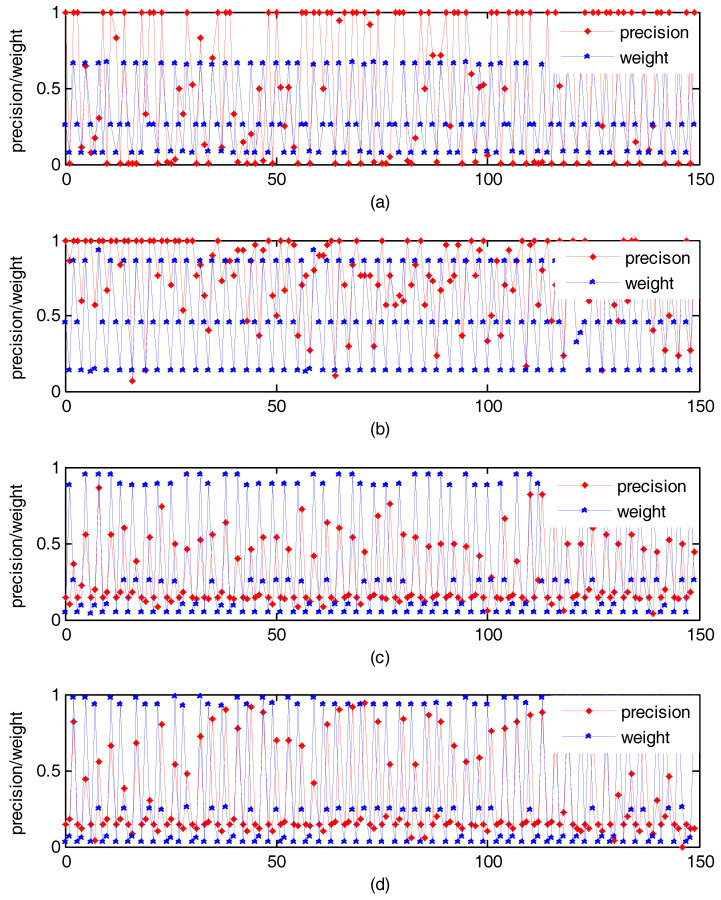
Under unsupervised condition, the change of weight obtained by our method with precision. (**a**) Experiment result on Holidays; (**b**) Experiment result on Wang; (**c**) Experiment result on UC Merced Land Use; (**d**) Experiment result on RSSCN7.

On Wang, UC Merced Land Use, RSSCN7, and Holidays, 50 images were randomly selected as query images, separately. We search similar images by our method. Figure 2 shows the change of weight with precision of each single feature. The abscissa is the features. From left to right, three points as 1 group, shows the precision and weights of each single feature of the same image retrieval. For example, in Figure 2a, the abscissa of 1–3 represents the three features of the first image in the 50 images selected from the Holidays. The blue line represents the weight, and the red line indicates the retrieval performance. We can see that the feature whose retrieval performance is excellent can obtain a relatively large weight by our method. That is to say, our method can make better use of good performance features, which is helpful to improve the retrieval performance.

**Figure 3:**
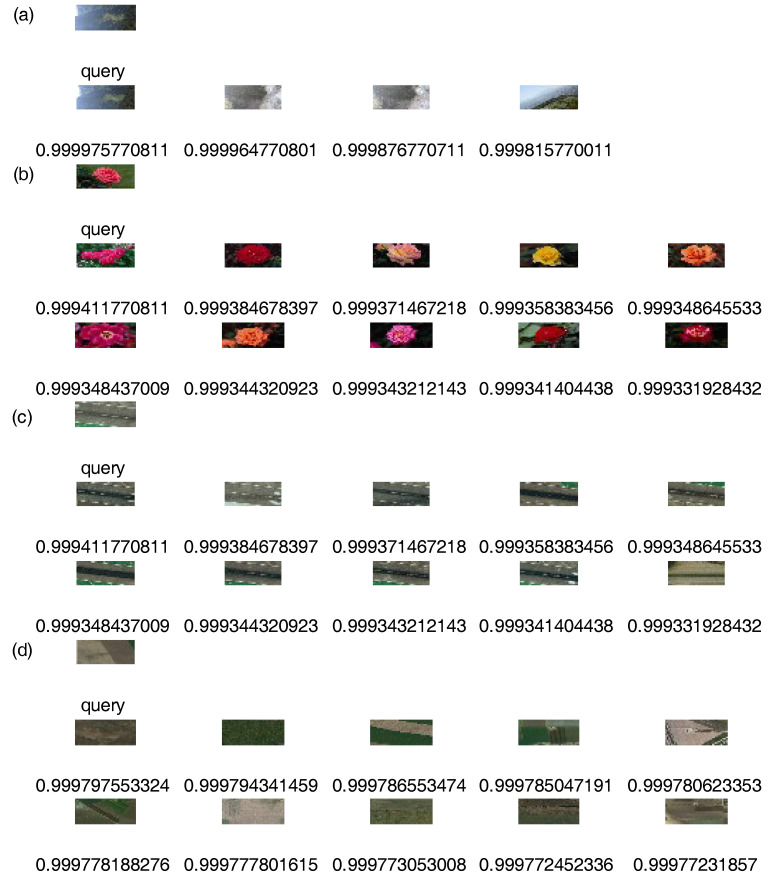
Under unsupervised condition, retrieval results were displayed. (**a**) Experiment result on Holidays; (**b**) Experiment result on Wang; (**c**) Experiment result on UC Merced Land Use; (**d**) Experiment result on RSSCN7.

On Wang, UC Merced Land Use, and RSSCN7, one image was randomly selected as a query image and Top 10 retrieval results obtained by our method, respectively. On Holidays, one image was randomly selected as query image, respectively, and the Top 4 retrieval results obtained by our method. Figure 3 shows the retrieval results. The first image in the upper left corner is a query image that is labeled “query”. The remaining images are the corresponding similar images that are labeled by a similarity measure such as 0.999. In accordance with similarity from large to small, we arrange retrieval results from left to right and from top to bottom. 

#### 4.3.2. Supervised

Under supervised conditions, in order to verify the effectiveness of the adaptive weight method proposed in this paper, we carried out experiments on Holidays, Wang, UC Merced Land Use, and RSSCN7 datasets. Table 3 shows a comparison of retrieval results based on RF and OURS. On the Holidays dataset, our method is better than RF to improve the retrieval precision by 0.26%. On the Wang dataset, our method improves the retrieval accuracy by 0.38% (Top 20), 0.38% (Top 30), and 0.34% (Top 50) compared with RF. On the UC Merced Land Use dataset, our method improves the retrieval accuracy by 0.38% (Top 20), 0.45% (Top 30), and 0.05% (Top 50) compared with RF. On the RSSCN7 dataset, our method improves the retrieval accuracy by 0.84% (Top 20), 0.84% (Top 30), and 0.63% (Top 50) compared with RF.

**Figure 4:**
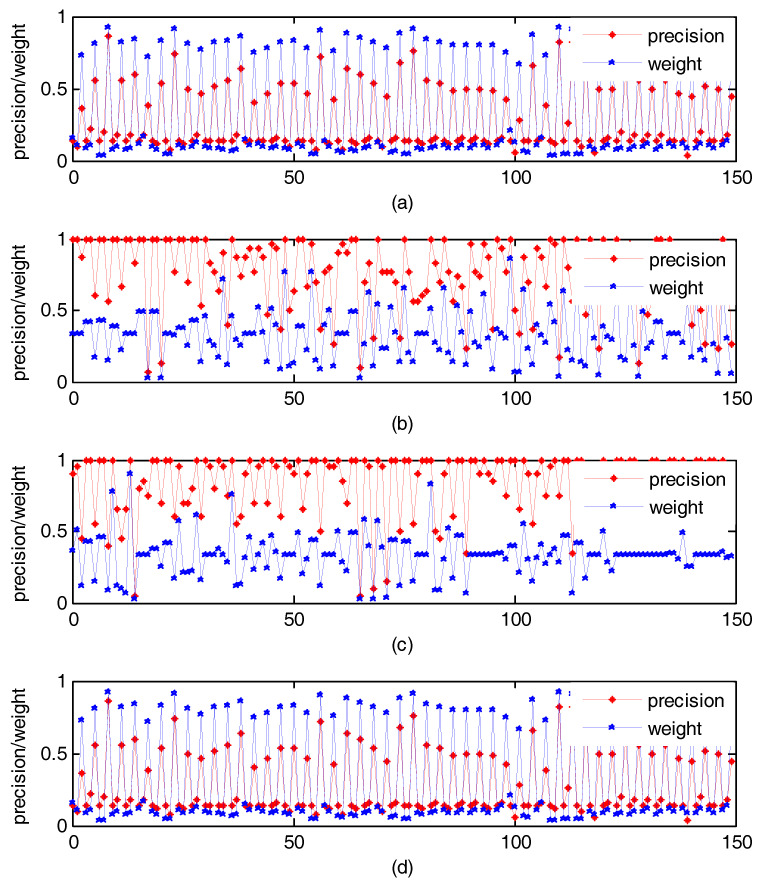
Under supervised condition, the change of weight that obtained by our method with precision. (**a**) Experiment result on Holidays; (**b**) Experiment result on Wang; (**c**) Experiment result on UC Merced Land Use; (**d**) Experiment result on RSSCN7.

Similar to unsupervised state, on Wang, UC Merced Land Use, RSSCN7, and Holidays, 50 images were randomly selected as query images, separately. We search similar images by our method. Figure 4 shows the change of weight with precision of each single feature. The abscissa is the features. From left to right, three points as 1 group, shows the precision and weight of each single feature of same image retrieval. For example, in Figure 2a, the abscissa 1–3 represents the three features of the first image in the 50 images selected from the Holidays. The blue line represents the weight and the red line indicates the retrieval performance. We can see that the retrieval performance of feature got by relevance feedback is excellent, and can obtain a relatively large weight by our method. That is to say, our method can make better use of good performance features, which is helpful to improve the retrieval performance.

**Figure 5:**
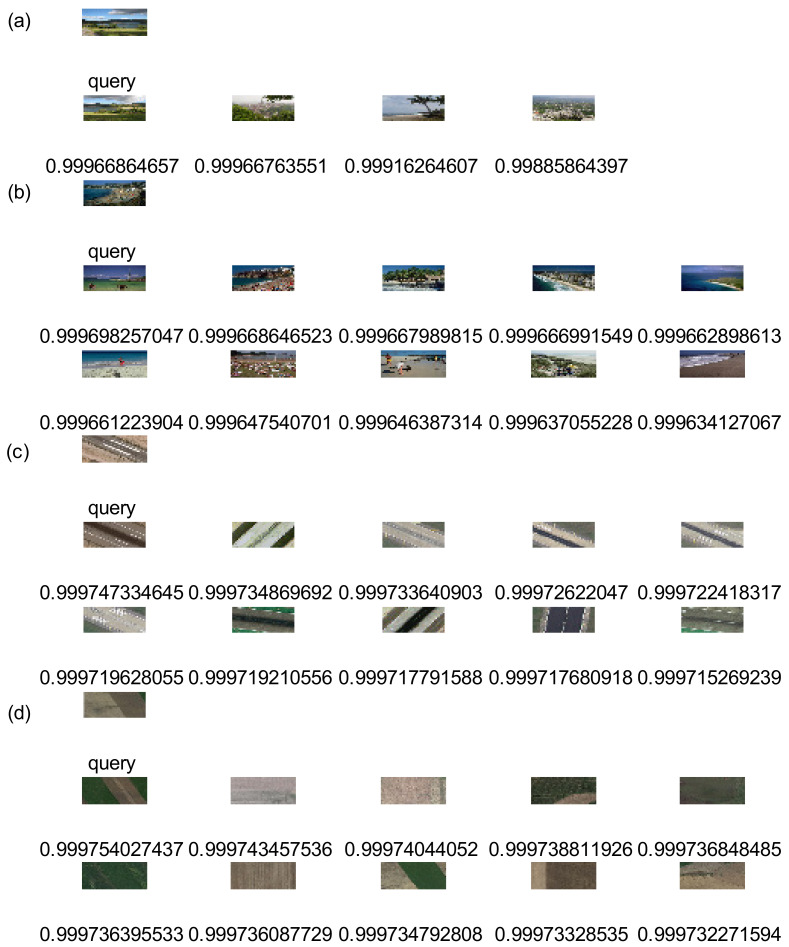
Under supervised condition, retrieval results were displayed. (**a**) Experiment result on Holidays; (**b**) Experiment result on Wang; (**c**) Experiment result on UC Merced Land Use; (**d**) Experiment result on RSSCN7.

Similar to unsupervised state, on Wang, UC Merced Land Use, RSSCN7, one image was randomly selected as query image, Top 10 retrieval results were obtained by through our method, respectively. On Holidays, one image was randomly selected as query image, respectively, Top 4 retrieval results obtained by our method. Figure 5 shows the retrieval results. The first image in the upper left corner is a query image that is labeled “query”. The remaining images are the corresponding similar images that are labeled by similarity measure such as 0.999. In accordance with similarity from large to small, we arrange them from left to right and from top to bottom. 

### 4.4. Comparison with Others Methods

In order to illustrate the performance of supervised and unsupervised methods compared with existing methods. In Table 4, we show the comparison results on the Wang dataset (Top 20). Under the state of unsupervision, the precision of our method is 97.09%, which is about 26% higher than previous methods listed [13,14]. Compared with a previous paper [12], it increased by approximately 9.26%. Compared with a previous paper [15], it increased by 24.42%. Compared with a previous paper [16], it increased by about 22.29%. Under the state of unsupervision, the precision of our method is 94.81%, which is about 23.72% higher than [13,14]. Compared with a previous paper [12], it increased by about 6.98%. Compared with a previous paper [15], it increased by 22.14%. Compared with a previous paper [16], it increased by about 20.01%. From the results, we can see that the method has achieved good results both under supervision and unsupervision. As suggested in Section 3, the supervised method requires users to participate in the feedback of single feature trust, which may cause some users’ aversion. The unsupervised method does not require users to participate in the selection of features, and directly outputs the retrieved images. The unsupervised method or supervised method is determined by the designer according to the actual use of the retrieval system. When we focus on user experience, we choose to be unsupervised. If we focus on higher retrieval accuracy, we choose to be supervised. After deciding whether to adopt supervised or unsupervised, the designer can make use of the corresponding solutions proposed in this paper to improve retrieval performance. 

Table 5 shows the comparison results on the Holidays dataset. The map of our method is 88.45%. Compared with a previous paper [7], it increased by about 1.55%. Compared with a previous paper [8], it increased by 2.93%. Compared with a previous paper [9], it increased by about 3.81%. Compared with a previous paper [10], it increased by about 0.45%. Compared with [17], it increased by about 9.15%. Compared with a previous paper [18], it increased by about 3.65%. 

(Note: To avoid misunderstanding, we do not use an abbreviation of each solution here, but the methods used in comparison are introduced in introduction.)

## 5. Discussion

Fusing multiple features can elevate the retrieval performance of retrieval system effectively. Meanwhile, in the process of multi-feature fusion, the proper single feature weight is helpful to further improve retrieval performance. This paper proposes a method to obtain single feature weights to fuse multiple features for image retrieval. 

Retrieval results on daily scene datasets, which are Holidays and Wang, and remote sensing datasets, which are UC Merced Land Use and RSSCN7, show that compared with single feature and fusing multiple features by averaging global weights and relevance feedback, our method has better retrieval performance. 

In the future work, there are two aspects of work that are worth doing. On the one hand, considering image retrieval based on multi-feature fusion increases the retrieval time; we will research how to improve the efficiency of retrieval. Many researches on image retrieval have been carried out on large-scale datasets, which may contain up to several million pictures, and it is very time-consuming to search for the images we need from the massive images. It is significant to improve the efficiency of retrieval. On the other hand, considering other forms of entropy have achieved good results in the image field [45,46], we will research other forms of entropy used in image retrieval. Meanwhile, considering the image decomposition and the classification of image patches has achieved outstanding results [47,48,49,50]. We can use the idea of image decomposition and the classification of image patches to extract better image description for retrieval system. It is significant to improve the performance of retrieval. 

## Figures and Tables

**Table 1:** Comparison of ways to determine weight.

Method	Pros	Cons
the global weight	short retrieval time	poor generalization performance/low retrieval performance
the adaptive weight	good generalization performance/excellent retrieval performance	long retrieval time

**Table 2:** Comparison of retrieval results based on AVG and OURS under unsupervised conditions.

Database	Holidays	Wang (Top)			UC Merced Land Use (Top)			RSSCN7 (Top)		
		20	30	50	20	30	50	20	30	50
AVG	0.7872	0.9446	0.9274	0.8924	0.8468	0.7851	0.6866	0.8842	0.8611	0.8251
OURS	0.8384	0.9481	0.9321	0.8982	0.9129	0.8784	0.8125	0.9103	0.8925	0.8635

**Table 3:** Comparison of retrieval results based on RF and OURS under supervised conditions.

Database	Holidays	Wang (Top)			UC Merced Land Use (Top)			RSSCN7 (Top)		
		20	30	50	20	30	50	20	30	50
RF	0.8819	0.9671	0.9539	0.9260	0.9247	0.8881	0.8250	0.9358	0.9191	0.8892
OURS	0.8845	0.9709	0.9577	0.9294	0.9285	0.8926	0.8255	0.9442	0.9275	0.8955

**Table 4:** Comparison with others methods on Wang.

Method	Ours		[11]	[12]	[13]	[14]	[15]	[16]
	Supervised	Unsupervised						
Africa	**87.70**	81.95	51.00	-	69.75	58.73	74.60	63.64
Beach	**99.35**	98.80	90.00	-	54.25	48.94	37.80	60.99
Buildings	**98.15**	97.25	58.00	-	63.95	53.74	53.90	68.21
Buses	**100.00**	100.00	78.00	-	89.65	95.81	96.70	92.75
Dinosaurs	**100.00**	100.00	100.00	-	98.7	98.36	99.00	100.00
Elephants	**99.10**	97.45	84.00	-	48.8	64.14	65.90	72.64
Flowers	99.95	99.45	**100.00**	-	92.3	85.64	91.20	91.54
Horses	**100.00**	100.00	100.00	-	89.45	80.31	86.90	80.06
Mountains	**98.00**	92.15	84.00	-	47.3	54.27	58.50	59.67
Food	**88.65**	81.05	38.00	-	70.9	63.14	62.20	58.56
Mean	**97.09**	**94.81**	78.3	87.83	70.58	70.31	72.67	74.80

**Table 5:** Comparison with others methods on Holidays.

Method	Ours	[7]	[8]	[9]	[10]	[17]	[18]
mAp	**88.45**	86.9	85.52	84.64	88.0	79.3	84.8
